# NRF2 Exerts Anti-Inflammatory Effects in LPS-Induced gEECs by Inhibiting the Activation of the NF-*κ*B

**DOI:** 10.1155/2021/9960721

**Published:** 2021-11-02

**Authors:** Hongchu Bao, Qinglan Qu, Wei Zhang, Xinrong Wang, Jianye Fang, Jinwen Xue, Zhenteng Liu, Shunzhi He

**Affiliations:** Department of Reproductive Medicine, Yantai Yuhuangding Hospital, Qingdao University, China

## Abstract

Nuclear factor E2-related factor 2 (NRF2) plays an anti-inflammatory role in several pathological processes, but its function in lipopolysaccharide- (LPS-) induced goat endometrial epithelial cells (gEECs) is still unknown. We designed a study to investigate the function of NRF2 in LPS-induced gEECs. LPS was found to increase the NRF2 expression and the nuclear abundance of NRF2 in gEECs in a dose-dependent manner. NRF2 knockout (KO) not only increased the expression of LPS-induced proinflammatory cytokines (TNF-*α*, IL-1*β*, IL-6 and IL-8) but also increased the expression of TLR4, p-I*κ*B*α*/I*κ*B*α*, and p-p65/p65 proteins. Immunoprecipitation experiments showed that NRF2 directly binds to p65 in the nucleus and inhibits the binding of p65 to downstream target genes (TNF-*α*, IL-1*β*, IL-6, and IL-8). Even though a NF-*κ*B/p65 inhibitor (PDTC) reduced the LPS-induced NRF2 expression and nuclear abundance of NRF2, overexpressing TNF-*α* reversed the inhibitory effects of PDTC on the NRF2 expression and on its abundance in the nucleus. Similarly, knockdown of the proinflammatory cytokines (TNF-*α*, IL-1*β*, IL-6, or IL-8) significantly decreased the LPS-induced NRF2 expression and NRF2 in the nucleus. In conclusion, our data suggest that proinflammatory cytokines induced by LPS through the TLR4/NF-*κ*B pathway promote the NRF2 expression and its translocation into the nucleus. Our work also suggests that NRF2 inhibits the expression of proinflammatory cytokines by directly binding to p65.

## 1. Introduction

After giving birth, animals often suffer from endometritis as a result of infection, making it difficult for them to have healthy pregnancies in the future as well as produce milk. Animals that have issues with pregnancies or milk production contribute to economic loss on farms [1]. In humans, bacterial infections can also cause endometritis and may lead to spontaneous abortion of cancer [2, 3]. There are many species of bacteria that cause endometritis, such as Escherichia coli, Arcanobacterium pyogenes, Prevotella spp., Fusobacterium necrophorum, and Fusobacterium nucleatum, most of which are gram-negative [4, 5]. Lipopolysaccharide (LPS) is a substance composed of lipids and polysaccharides that composes the outer cell wall of gram-negative bacteria and reported as the main toxic factor of gram-negative bacteria causing an immune response [6, 7]. Therefore, in the study presented here, LPS was used to simulate infection by gram-negative bacteria.

The uterine immune response caused by gram-negative bacteria results not only from immune cells, such as macrophages, T cells, and granulocytes, but also from inherent uterine cells, such as endometrial epithelial cells and stromal cells. Endometrial cells also express receptors that recognize microbial pathogen-associated molecular patterns and lead to the production of inflammatory cytokines, which is one of the pathological mechanisms leading to endometrial inflammation [8, 9]. Therefore, preventing inflammation caused by endometrial cells may also help resolve endometritis.

Nuclear factor E2-related factor (NRF2) is an important transcription factor in the CNC family that plays an anti-inflammatory role in the inflammatory response [10, 11]. Previous studies found that NRF2 exerts anti-inflammatory effects in LPS-treated mouse peritoneal macrophages [12, 13], microglia [14], and in response to mouse kidney and lung injuries [15, 16]. Usually, the TLR4/NF-*κ*B signaling pathway is examined when studying NRF2 inhibition of LPS-induced inflammation, since the toll-like receptor 4 (TLR4) recognizes LPS and is expressed by immune and ordinary cells [17, 18], including endometrial epithelial cells [8, 9]. However, the function of NRF2 in LPS-treated endometrial epithelial cells is still unclear. In this study, we determined the function of NRF2 in LPS-induced gEECs and explored its relationship with the TLR4/NF-*κ*B signaling pathway.

## 2. Materials and Methods

### 2.1. Cell Culture and Reagents

The study protocol was approved by the Medical Ethics Committee of Yantai Yuhuangding Hospital, conformed to the Principles of Laboratory Animal Care (National Society for Medical Research), and was conducted according to National Institutes of Health guidelines.

Immortalized gEECs were established as previously described [19]. Briefly, anesthesia was induced using 5 mg/kg propofol administered through rapid injection into the cephalic vein and maintained with halothane in 100% oxygen. A vaporizer was adjusted to maintain ETHAL between 0.95% and 1.0% [20, 21]. Next, goats received cuts to their necks for euthanasia according to the halal slaughter procedure (HS) as outlined in the MS1500:2009 (Department of Standards Malaysia 2009) [22] and “Guide for the Care and Use of Laboratory Animals: Eight Edition” from USA [21]. Goats were confirmed dead when it was observed that they no longer had a heartbeat nor were breathing. Once confirmed dead, endometrium samples were harvested [21]. All endometrium samples were briefly washed using phosphate buffered saline (PBS) and then immediately used to isolate gEECs using digestion with trypsin. gEECs were cultured in Dulbecco's Modified Eagle Medium/Nutrient Mixture F-12 medium (DMEM/F12, Gibco, USA) containing 10% fetal bovine serum (FBS,Gibco, USA) at 37°C with 5% CO_2_. 293 T cells were purchased from American Type Culture Collection and cultured in DMEM medium (Gibco, USA) containing 10% fetal bovine serum (FBS,Gibco, USA) at 37°C with 5% CO_2_. We seeded 6 × 10^6^ gEECs in a 10 cm cell culture dish and after 24 hours, different concentrations of lipopolysaccharide (LPS, sigma, USA) were added for 12 hours. Pyrrolidinedithiocarbamate ammonium (PDTC), an NF-*κ*B inhibitor, was purchased from Sigma; 50 *μ*mol/L of PDTC and 8 *μ*g/mL of LPS were added to gEECs culture at the same time for 12 hours.

### 2.2. RT-qPCR Analysis

Cells were harvested after being processed, and the total RNA from the cells was extracted using the RNA extraction kit (Solarbio, China). Next, cDNA was prepared from RNA using a reverse transcription kit (TARAKA, Japan), and 20 *μ*l of qPCR master mix was prepared and analyzed as described by the manual provided with the GoTaq qPCR Master Mix (Promega, USA). The relative expression of mRNA was calculated using the 2^-*ΔΔ*Ct^ method, and *β*-actin was used as the reference control. Primer sequences are shown in Table 1.

### 2.3. Immunoblotting

Total protein was extracted from cells using Nuclear/Cytosol Fractionation Kit (Biovision, USA) plus protease and phosphatase inhibitors (Solarbio, China). A BCA kit (ThermoFisher, USA) was used to determine the protein concentration. Next, 40 *μ*g of total protein was fractionated by SDS-PAGE on a 10% gel. After the transfer, the PVDF membrane (ThermoFisher, USA) was first blocked with 5% skimmed milk solution, and then probed with primary antibodies against NRF2 (D1Z9C), TLR4, p-I*κ*B*α*, I*κ*B*α*, p-p65, and p65. Proteins were visualized using ECL solution (Beijing Xinjingke Biotechnologies Co., Ltd., China), followed by densitometry analysis using ImageJ 3.0 (IBM, USA); *β*-actin was used as a control. All antibodies used for immunoblotting were purchased from Cell Signaling Technology and were diluted according to manufacturer's instructions.

### 2.4. TNF-*α*, IL-1*β*, IL-6, and IL-8 ELISA Assays

We harvested the cell culture medium after treatment with 8 *μ*g/mL of LPS for 12 hours and centrifuged the medium (1000 g, room temperature) to pellet debris. The concentrations of TNF-*α*, IL-1*β*, IL-6, and IL-8 in the cell culture supernatant were determined using the enzyme-linked immunosorbent assay kit (Shanghai Kanglang Biotechnology Co., Ltd., China).

### 2.5. Immunofluorescence

Cells were fixed with 4% paraformaldehyde for 15 minutes at room temperature. After blocking with 5% BSA in 0.3% Triton X-100 for 1 hour at room temperature, a NRF2 (D9J1B) antibody was added. All slides were incubated with 5 *μ*g/mL of DAPI for 5 minutes at room temperature to counterstain the nucleus. Slides were analyzed using a Leica TCS SP5 microscope (Leica microsystem) with the LAS AF Lite 4.0 image browser software.

### 2.6. NRF2 Knockdown and Knockout

The CRISPR-Cas9 technique was used to knockout the NRF2 gene. Briefly, the specific gRNA for NRF2 (oligo 1: 5′-CACCGCAGCTGGATCTTCCGCTCAA-3′, oligo 2: 5′-AAACTTGAGCGGAAGATCCAGCTGC-3′) was bound to the lentiCRISPRv2 vector (Addgene, USA) and was transfected into 293 T cells (ATCC, USA) with psPAX2 (Addgene, USA) and pND2.G (Addgene, USA) plasmids using HG-trans293 (HealthGene, Canada) according to the manufacturer's instructions. After 72 hours, we collected 293 T cell culture supernatant containing lentivirus and added it into gEECs cell culture medium. After 72 hours, 1 *μ*g/mL of puromycin (Sigma, USA) was added to gEECs culture to select for NRF2 KO cells.

For knockdown studies, we directly transfected 50 nmol/l of small interfering RNAs (siRNAs) into 2.5 × 10^6^ gEECs using Lipofectamine 2000 (ThermoFisher, USA) according to the manufacturer's protocol. After 72 hours, we performed subsequent experiments. The sequences of the siRNAs used in this study are shown in Table 1.

### 2.7. Immunoprecipitation and Pull-down Assays

Immunoprecipitation (IP) was used to analyze protein-protein binding. Briefly, cells were lysed using IP lysis buffer (LEAGENE, China) or Nuclear/Cytosol Fractionation Kit (Biovision, USA) to extract proteins. We added 1 *μ*g of NRF2 (D1Z9C) or p65 (D14E12) antibodies (Cell Signaling Technology, USA) into 200 *μ*g protein from cells for 12 hours at 4°C. Next, 100 *μ*l of protein A/G beads (Santa Cruz Biotechnology) was added into the mixture for 12 hours at 4°C. Lastly, we collected the beads by centrifuging (1000 g, 4°C); the beads were boiled to dissociate the proteins, and protein identification was performed by immunoblotting.

### 2.8. Chromatin Immunoprecipitation

Chromatin immunoprecipitation (ChIP) was used to analyze the dynamics of p65 binding at the promoter region of proinflammatory cytokines (TNF-*α*, IL-1*β*, IL-6, and IL-8), as previously described [23]. Briefly, cells were lysed using SDS lysate after crosslinking with 1% formaldehyde at room temperature for 10 minutes and then sonicated to obtain DNA fragments. We harvested the sonicated supernatant after centrifuging (1000 g, room temperature) and mixed it with 9 volumes of ChIP dilution buffer. Next, half volumes of protein-A agarose were added into the mixture at 4°C for 30 minutes. Chromatin was immunoprecipitated overnight at 4°C with 5 *μ*g of NF-*κ*B p65 (D14E12) antibody (Cell Signaling Technology, USA). Lastly, the precipitated DNA was analyzed using the promoter-specific PCR primer pairs (Table 1).

### 2.9. Statistical Analysis

Statistical Product and Service Solutions 20.0 (IBM, USA) software was used to analyze the data in the present study. The difference between two groups was analyzed by Student's *t*-test; one-way ANOVA with Tukey's test as a posthoc test was used to analyze the difference between multiple groups. *P* value less than 0.05 indicated significant differences.

## 3. Results

### 3.1. LPS Increased NRF2 Expression and Nuclear Abundance

**Figure 1:**
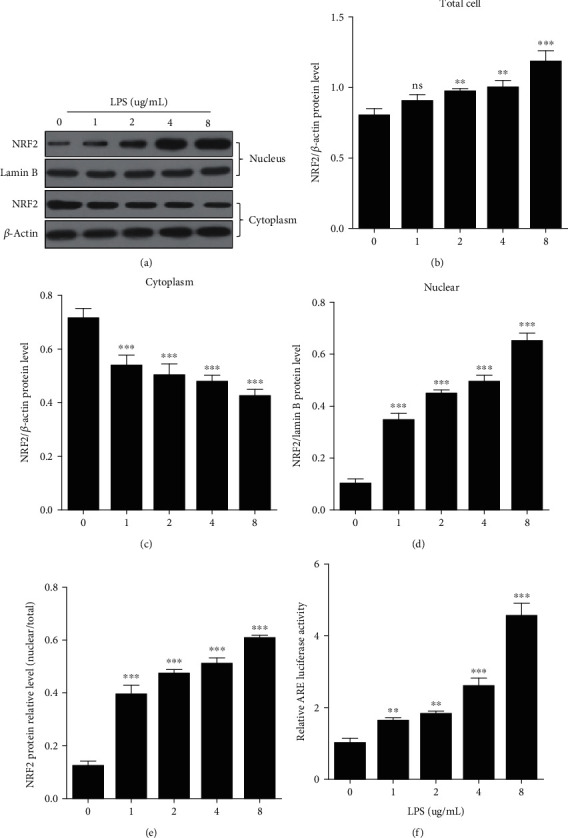
Induction of NRF2 in gEECs induced by LPS. GEECs were exposed to different concentrations of lipopolysaccharide (LPS) for 12 hours and then detected the expression of NRF2 protein by immunoblotting. (a) Representative NRF2 protein band was quantified in (b) total cells, (c) cytoplasm, (d) nucleus, and (e) the ratio of nuclear/total cells, and we also detected ARE luciferase activity (f). Data shown are mean ± SD, and ns was *P* > 0.05, ^∗∗^ was *P* < 0.01, and ^∗∗∗^ was *P* < 0.001 vs. LPS = 0 *μ*g/mL. *P* value was calculated by the one-way ANOVA with Tukey's test as a posthoc test.

After treatment with different concentrations of lipopolysaccharide (LPS, 0, 1, 2, 4, and 8 *μ*g/mL) for 12 hours, we harvested the gEECs to extract cytoplasmic and nuclear proteins. Immunoblotting was used to detect the expression of NRF2; lamin B was loaded as the nucleus control, and *β*-actin was loaded as the cytoplasm control (Figure 1(a)). The analysis of the gray value of protein bands showed that the LPS -increased expression of NRF2 in gEECs in a dose-dependent manner (Figure 1(b)). In the cytoplasm, LPS induced the decreased expression of NRF2 in gEECs in a dose-dependent manner (Figure 1(c)). However, in the nucleus, the LPS-induced increased expression of NRF2 in a dose-dependent manner (Figures 1(d) and 1(e)). In addition, we also detected the luciferase activity of ARE to show the effects of LPS stimulation on the DNA binding activity of NRF2 in gEECs cells and found that LPS induced luciferase activity of ARE in gEECs in a dose-dependent manner (Figure 1(f)).

**Figure 2:**
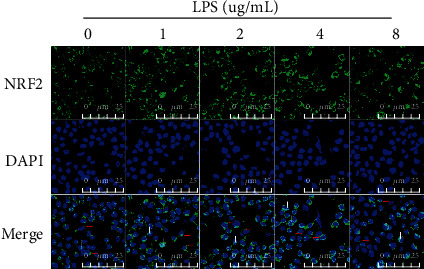
LPS increased nuclear abundance of NRF2 in gEECs. GEECs are exposed to different concentrations of LPS for 12 hours, and then the dynamic changes of cytoplasmic and nuclear NRF2 was imaged by immunofluorensent staining. Scale bar was 45 *μ*m. The red arrow indicates NRF2 located in the cytoplasm, and the white arrow indicates NRF2 located in the nucleus.

To test the nuclear abundance of NRF2 induced by LPS, we used immunofluorescence staining to observe the dynamic changes of cytoplasmic and nuclear NRF2 and found that (Figure 2) NRF2 was exclusively stained in cytoplasm in gEECs at basal status. In fact, NRF2 was clearly expressed in the nucleus of the control group stimulated with 0 *μ*g/mL of LPS, but was expressed at lower levels in the treatment groups. NRF2 was predominantly stained in the nucleus after LPS stimulation in a dose-dependent manner, which was consistent with immunoblots.

### 3.2. NRF2 Knockout Increased LPS-Induced Inflammation

**Figure 3:**
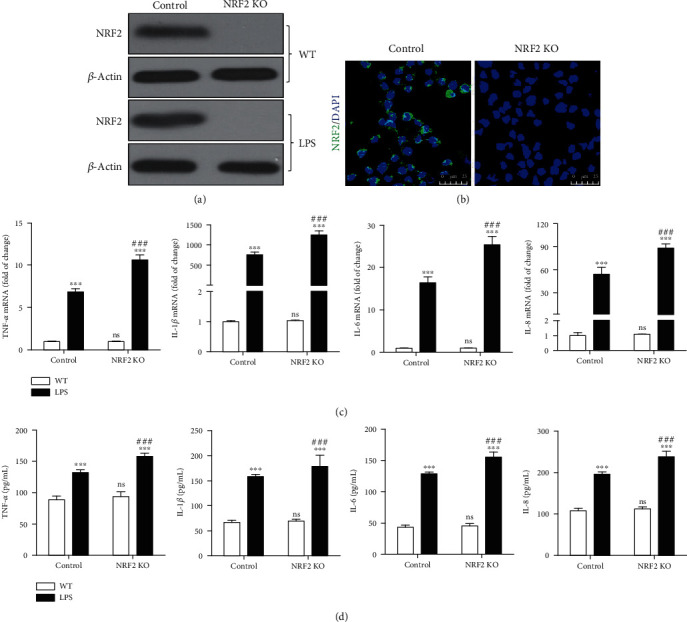
Depleting NRF2 increased LPS-induced proinflammatory cytokines expression. Control and NRF2 knockout (KO) gEECs were stimulated with or without 8 *μ*g/mL LPS for 12 hours and then (a) detected the NRF2 protein expression by immunoblotting. (b) The dynamic changes of cytoplasmic and nuclear NRF2 were imaged by immunofluorensent staining. (c) RT-qPCR analysis of the indicated TNF-*α*, IL-1*β*, IL-6, and IL-8 mRNA expression. (d) The concentrations of proinflammatory cytokines (TNF-*α*, IL-1*β*, IL-6, and IL-8) in culture medium were determined with ELISA. Data shown are mean ± SD; ns was *P* > 0.05, and ### was *P* < 0.001 vs. control group; ^∗∗∗^ was *P* < 0.001 vs. WT group. *P* value was calculated by the one-way ANOVA with Tukey's test as a posthoc test. Scale bar was 45 *μ*m.

Since LPS is usually associated with inflammation, we hypothesized that NRF2 exerted the regulation of inflammation in LPS-treated gEECs. To study the function of NRF2, we knocked out the NRF2 expression using the CRISPR-Cas9 technique. The results of immunoblotting (Figure 3(a)) and immunofluorescence staining (Figure 3(b)) showed that we successfully established NRF2 knockout in gEECs. Next, after treatment with 8 *μ*g/mL of LPS for 12 hours, we harvested the cells and culture medium. Total RNA was extracted from cells, and RT-qPCR was used to determine the mRNA expression of proinflammatory cytokines (TNF-*α*, IL-1*β*, IL-6, and IL-8). As shown in Figure 3(c), the LPS increased mRNA expression of proinflammatory cytokines (TNF-*α*, IL-1*β*, IL-6, and IL-8). Importantly, although NRF2 knockout alone did not increase the expression of proinflammatory cytokines, NRF2 knockout increased the expression of proinflammatory cytokines with 8 *μ*g/mL of LPS stimulation. Similarly, compared with normal gEECs, the concentration of proinflammatory cytokines (TNF-*α*, IL-1*β*, IL-6, and IL-8) in the culture medium of NRF2 KO gEECs was higher (Figure 3(d)). In addition, we also detected some proinflammatory or anti-inflammatory-related genes, such as iNOS, COX-2, HO-1, and NQO1, and found that (Figure S1) LPS increased the mRNA expression of proinflammation genes (iNOS and COX-2) and anti-inflammation genes (HO-1 and NQO1), but NRF2 knockout significantly increased the expression of proinflammation genes (iNOS and COX-2) and decreased the expression of anti-inflammation genes (HO-1 and NQO1) with 8 *μ*g/mL of LPS stimulation.

### 3.3. NRF2 Knockout Activated TLR4/NF-*κ*B Pathway

**Figure 4:**
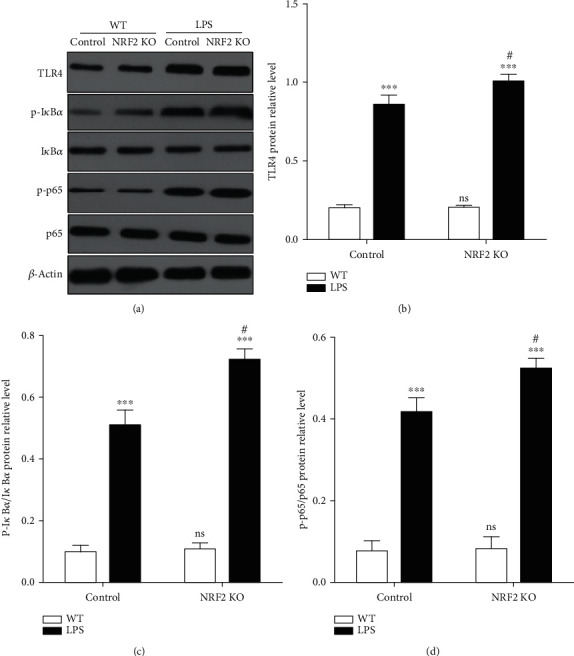
Depleting NRF2 increased LPS-induced the activation of TLR4/NF-*κ*B pathway. Control and NRF2 knockout (KO) gEECs were stimulated with or without 8 *μ*g/mL LPS for 12 hours and (a) then detected protein expression by immunoblotting and quantified TLR4 (b), p-I*κ*B*α*/I*κ*B*α* (c), and p-p65/p65 (d) protein expression. Data shown are mean ± SD; ns was *P* > 0.05, and # was *P* < 0.05 vs. control group; ^∗∗∗^ was *P* < 0.001 vs. WT group. *P* value was calculated by the one-way ANOVA with Tukey's test as a posthoc test.

TLR4/NF-*κ*B pathway plays an important role in LPS-induced inflammation. LPS interacts with TLR4 and binds to LBP-LPS-CD14, which promotes intracellular signals and promotes the phosphorylation, ubiquitination, and degradation of I*κ*B*α* to release NF-*κ*B. This in turn initiates an inflammatory response that activates a series of downstream molecules (including TNF-*α*, IL-1*β*, IL-6, and IL-8) that amplify the inflammatory response [24]. To test the effects of NRF2 on the activation of the TLR4/NF-*κ*B pathway, we assessed the expression of important mediators such as TLR4, I*κ*B*α*, and NF-*κ*B p65. The results showed that LPS activates the TLR4/NF-*κ*B pathway in normal gEECs, and that LPS increased the expression of TLR4, p-I*κ*B*α*/I*κ*B*α*, and p-p65/p65 proteins. However, no significant differences were observed in the expression levels of I*κ*B*α* and p65 (Figure 4(a)). Importantly, the expression levels of TLR4 (Figure 4(b)), p-I*κ*B*α*/I*κ*B*α* (Figure 4(c)), and p-p65/p65 (Figure 4(d)) were significantly higher in NRF2 KO gEECs than in normal gEECs.

### 3.4. NRF2 Interacts with p65 in the Nucleus and Inhibited the DNA-Binding Activity of p65

**Figure 5:**
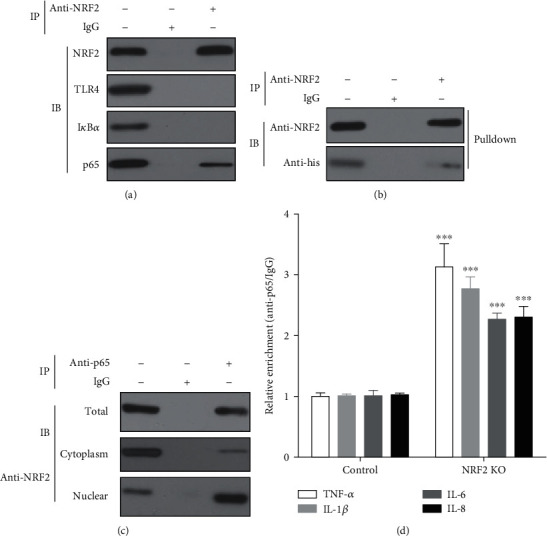
NRF2 binds to p65 in the nucleus, hindering the DNA binding activity of p65. GEECs were harvested and prepared cell lysis after treating with 8 *μ*g/mL LPS for 12 hours. (a) Immunoprecipitation with NRF2 antibody (Rabbit IgG was used as negative control) and immunoblotting with NRF2, TLR4, I*κ*B*α,* and p65 antiboty. (b) Prokaryotic expression of NRF2 protein with his label was incubated with protein A/G beads coprecipitated with NRF2 antibody for 12 hours and immunoblotting with NRF2 and his antibody. (c) Immunoblotting with NRF2 antibody, and (d) ChIP assays were performed with IgG and anti-p65 antibody; ChIP values are shown as fold changes of immunoprecipitated promoter fragments over IgG controls. ^∗∗∗^ was *P* < 0.001 vs. control group, and *P* value was calculated by Student's *t*-test.

To study the mechanisms behind the effects of NRF2 on the TLR4/NF-*κ*B pathway, we used a NRF2 antibody through immunoprecipitation experiments to find that only NRF2 and p65 proteins were detected among the proteins binding toNRF2 (Figure 5(a)). Moreover, to confirm that the interaction between p65 and NRF2 was direct, we used the p65 protein expressed in prokaryotic cells for pull-down experiments. The results showed that (Figure 5(b)) the interaction between p65 and NRF2 was direct. However, we noticed that although the results of immunoprecipitation showed that p65 can bind to NRF2, the enrichment results of the NRF2 antibody to p65 were not observed. In contrast, the amount of p65 protein bound by NRF2 was lower than in the cell lysate (Figure 5(a)), which indicated that NRF2 only binds to p65 at a certain location. Therefore, we isolated nuclear and cytoplasmic proteins to assess the binding of NRF2 to p65 using immunoprecipitation with a p65 antibody and found that (Figure 5(c)) NRF2 was bound with p65 in the nucleus. Further, we performed ChIP-qPCR to investigate whether NRF2 regulates the expression of downstream target genes by affecting the binding of p65 to the target gene promoter and found that NRF2 KO significantly improves the binding activity of p65 to the promoter regions of TNF-*α*, IL-1*β*, IL-6, and IL-8 (Figure 5(d)).

### 3.5. LPS-Induced NRF2 Depends on Proinflammatory Cytokines

**Figure 6:**
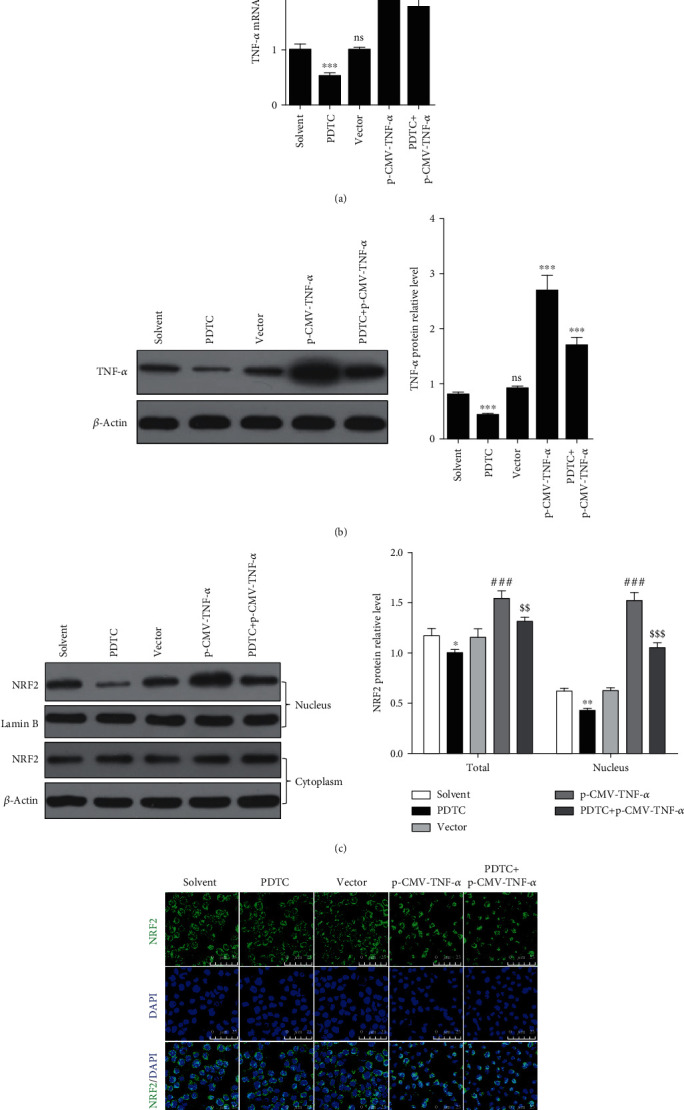
LPS-induced NRF2 expression and nuclear abundance of NRF2 depended on TNF-*α*. Control and TNF-*α* overexpression (p-CMV- TNF-*α*) gEECs were exposed to 50 *μ*mol/L PDTC and 8 *μ*g/mL LPS for 12 hours. (a) RT-qPCR analysis was used to detect the expression of TNF-*α* mRNA. Data shown are mean ± SD, ns was *P* > 0.05, and ^∗∗∗^ was *P* < 0.001 vs. solvent group. *P* value was calculated by the one-way ANOVA with Tukey's test as a posthoc test. (b) Immunoblotting was used to detect the expression of TNF-*α* and (c) NRF2 expression, and we harvested nuclear and cytoplasmic total proteins separately to detect the expression of NRF2 protein; so, the sum of NRF2 expression in nucleus and cytoplasm is the expression level in total cells. Data shown are mean ± SD, ^∗^ was *P* < 0.05, and ^∗∗^ was *P* < 0.01 vs. solvent group, ### was *P* < 0.001 vs. vector group, $$ was *P* < 0.01, and $$$ was *P* < 0.001 vs. p-CMV-TNF-*α* group. *P* value was calculated by Student's *t*-test. (d) The dynamic changes of cytoplasmic and nuclear NRF2 were imaged by immunofluorensent staining. Scale bar was 45 *μ*m.

Previous studies have shown that NF-*κ*B inhibits NRF2 in multiple ways. For example, NF-*κ*B/p65 antagonizes the Nrf2-ARE pathway by depriving CBP from Nrf2 and facilitating the recruitment of HDAC3 to MafK [25]. Additionally, nuclear factor p65 interacts with Keap1 to repress the Nrf2-ARE pathway [26]. Therefore, we sought to identify what mechanisms were necessary for the induction of NRF2 in gEECs treated with LPS. We hypothesized that NF-*κ*B/p65 or its downstream molecules played an important role in the induction of NRF2 induced by LPS. To test this, we used a NF-*κ*B/p65 inhibitor, pyrrolidinedithiocarbamate ammonium (PDTC), to inhibit NF-*κ*B/p65 and established TNF-*α* overexpressing gEECs by performing transfection with p-CMV-TNF-*α*. First, RT-qPCR analysis indicated that PDTC significantly decreased the LPS-induced TNF-*α* mRNA expression, but transfection with p-CMV-TNF-*α* successfully increased the TNF-*α* mRNA expression. In addition, the expression of TNF-*α* mRNA in TNF-*α*-overexpressing gEECs with PDTC stimulation was significantly lower than in the solvent control group (Figure 6(a)). The same change was also found in the TNF-*α* protein levels detected using immunoblotting (Figure 6(b)). Interestingly, PDTC not only significantly reduced the overall expression levels of NRF2 but also reduced the expression levels of NRF2 in the nucleus (Figure 6(c)), which was inconsistent with previous studies [25, 26]. However, overexpressing TNF-*α* increased the NRF2 expression in the cells and in the nucleus, and PDTC could not reduce the NRF2 protein expression and the nuclear abundance of NRF2 in gEECs overexpressing TNF-*α*, compared with the solvent (control) group (Figures 6(c) and 6(d)).

**Figure 7:**
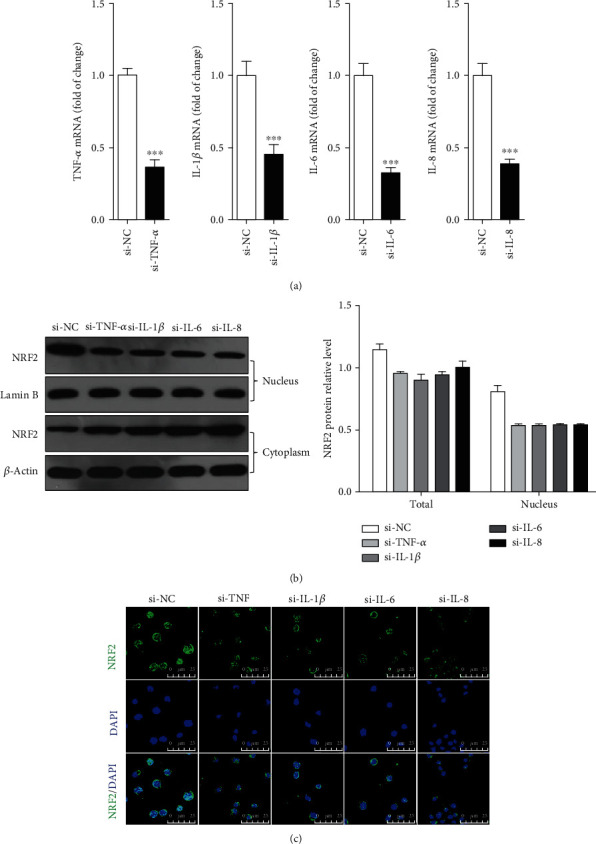
Depleting proinflammatory cytokine expression decreased LPS-induced NRF2 expression and nuclear abundance of NRF2. 72 hours after transferring si-RNA to gEECs, (a) RT-qPCR was used to detect the expression of TNF-*α*, IL-1*β*, IL-6, and IL-8 mRNA. (b) Immunoblotting was used to detected the expression of NRF2. (c) The dynamic changes of cytoplasmic and nuclear NRF2 were imaged by immunofluorensent staining.^∗∗∗^ was *P* < 0.001 vs. si-NC group, and *P* value was calculated by the one-way ANOVA with Tukey's test as a posthoc test. Scale bar was 45 *μ*m.

**Figure 8:**
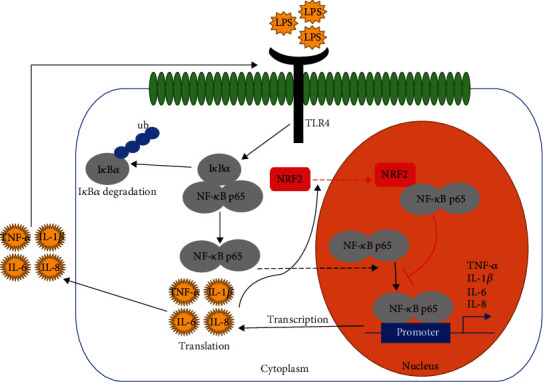
NRF2 exerts anti-inflammatory effects in LPS-induced gEECs via TLR4/NF-*κ*B pathway. Graphic description of this study. Proinflammatory cytokines induced by LPS through the TLR4/NF-*κ*B pathway promoted NRF2 expression and transferred into the nucleus, and the nucleus NRF2 inhibited the expression of proinflammatory cytokines by directly binding to p65.

These results indicated that LPS-induced NRF2 expression and nuclear abundance of NRF2 was dependent upon TNF-*α*, not NF-*κ*B/p65. To test other proinflammatory cytokines, we transfected respective small interfering RNA (siRNA) into gEECs to inhibit proinflammatory cytokine expression. RT-qPCR results showed that we successfully established TNF-*α*, IL-1*β*, IL-6, or IL-8 knock down in gEECs (Figure 7(a)). Immunoblotting results indicated that knockdown of proinflammatory cytokines (TNF-*α*, IL-1*β*, IL-6, or IL-8) significantly decreased the NRF2 expression and nuclear abundance of NRF2 (Figures 7(b) and 7(c)). These results suggested that proinflammatory cytokines induced by LPS through the TLR4/NF-*κ*B pathway promoted the NRF2 expression, which then translocated to the nucleus and inhibited the expression of proinflammatory cytokines by directly binding to p65 (Figure 8).

## 4. Discussion

Endometritis is not only a disease that affects humans but also one of the primary problems facing the livestock industry. Statistics show that 14-53% of cows and 10–20% of mares could develop endometritis due to various infections [27, 28]. Importantly, animals with endometritis have prolonged birth cycles, decreased milk production, and delayed growth, which is dangerous to the livestock industry [1]. Therefore, it is important to explore the pathological mechanisms of endometritis. Endometrial epithelial cells have been confirmed by many studies as an effective model system for studying endometrial diseases [7, 19] and are considered to be the body's inherent immune system against the invasion of foreign microorganisms [29]. LPS is the main toxic molecule of gram-negative bacteria and has been used to simulate the gram-negative bacteria infection in vivo and in vitro for many years [7, 19]. Here, we established an LPS-induced endometrial epithelial cell inflammation model in vitro to study the function of NRF2 in innate immunity. Even though the working concentration of LPS is usually 100-2000 ng/mL, we found that LPS increased the NRF2 expression and nuclear abundance of NRF2 in a dose-dependent manner. The proper concentration of LPS may be determined based on the specific research study [20, 22], but the working concentration of LPS is 8 *μ*g/mL in this study. NRF2 is an important transcription factor in the CNC family, which is located at the 2q31 locus. It has six different functional regions named Neh1-Neh6, which are widely present in various tissues and organs and interact with antioxidant elements ARE to activate downstream gene transcription to further regulate antioxidant and anti-inflammatory proteins [30]. In this study, we found that after LPS treatment, the overall expression levels of NRF2 and its nuclear abundance increased in a dose-dependent manner. NRF2 knockout increased the expression of proinflammatory cytokines (TNF-*α*, IL-1*β*, IL-6, and IL-8) in LPS-treated gEECs. Previous studies found that NRF2 exerts anti-inflammatory functions in many LPS-treated cells as well as in animal models [31, 32]. For instance, activated AMP-activated protein kinase (AMPK) inhibits the expression of LPS-induced inflammatory genes by promoting nuclear translocation and phosphorylation of NRF2, thereby improving the survival rate of LPS-treated mice [31]. In addition, NRF2 is a target for many drugs to treat LPS-induced inflammation. Lee et al. found that 3,4,5-trihydroxycinnamic acid inhibits lipopolysaccharide- (LPS-) induced inflammation by NRF2 activation in vitro and improves survival of mice in LPS-induced endotoxemia model in vivo [33]. Qi et al. found that isorhamnetin could decrease the LPS-induced expression of PGE2, NO, IL-6, and IL-8 in human gingival fibroblasts, but NRF2 knockdown reversed the anti-inflammatory effects of isorhamnetin, which indicated that NRF2 was necessary for isorhamnetin to inhibit LPS-induced inflammation in human gingival fibroblasts [34]. Therefore, if NRF2 plays an anti-inflammatory role in the LPS-induced endometrial epithelial cell inflammation model; then, those drugs that have been confirmed to target NRF2 can be used as alternative drugs for the treatment of endometritis. For molecular mechanisms, previous studies have found that NRF2 inhibits LPS-activated inflammation by inhibiting the HO-1-mediated p65-p300 signaling pathway in BV2 microglia [35], and inhibition of NRF2 can reduce kidney inflammation in mice by limiting the activation of oxidative stress and NF-*κ*B signaling pathways [36]. Although these previous studies are not in gEECs, they have reference significance for this study, such as the fact that inhibition of NRF2 suppresses the NF-*κ*B signaling pathway in vivo. The TLR4/NF-*κ*B signaling pathway is an important effector pathway for LPS-induced inflammation. When LPS is released into the circulatory system by invading microorganisms, it binds to TLR4 expressed on the cell membrane and then creates intracellular signals to initiate an inflammatory response that activates a series of downstream molecules that further amplify the inflammatory response [24]. The NF-*κ*B pathway is an important downstream pathway of the LPS-TLR4 pathway. The activated TLR4 signaling pathway can release NF-*κ*B through ubiquitination and phosphorylation of I*κ*B*α*, following which NF-*κ*B enters the nucleus through phosphorylation or acetylation modification [24]. Lastly, NF-*κ*B in the nucleus combines with targeted DNA to promote the expression of proinflammatory cytokines, such as TNF-*α*, IL-1*β*, IL-6, IL-8, IL-10, and IL-12 [37, 38]. These cytokines not only directly lead to inflammation but also activate other signaling pathways (include TLR4) and promote the expression of inflammatory factors, thereby magnifying the inflammatory response [24]. Therefore, we investigated the effects of the NRF2 expression on the activation of the TLR4/NF-*κ*B pathway in LPS-induced gEECs and found that the expression of TLR4, p-I*κ*B*α*/I*κ*B*α*, and p-p65/p65 was significantly higher in NRF2 KO gEECs than in normal gEECs, which indicated that NRF2 inhibited the TLR4/NF-*κ*B pathway in LPS-induced gEECs.

To further investigate the mechanisms of action of NRF2 on the TLR4/NF-*κ*B pathway, immunoprecipitation studies were used to analyze the role of important molecules in the NRF2 and TLR4/NF-*κ*B pathway and found that only p65 protein binds to NRF2 protein. The following pull-down experiment also showed that NRF2 directly binds to p65 in the nucleus. However, we found that NRF2 does not affect the abundance of the p65 protein expression in LPS-induced gEECs. We hypothesized that NRF2 affected the DNA binding activity of NF-*κ*B p65. This was confirmed by data from subsequent experiments. Previous studies have found that NF-*κ*B inhibited NRF2 by depriving CBP from NRF2 and facilitating the recruitment of HDAC3 to MafK [25],or by interacting with Keap1 [26], which seems inconsistent with our results. Therefore, we suppressed NF-*κ*B using a NF-*κ*B inhibitor (PDTC), but found that PDTC could reduce the LPS-induced NRF2 expression and the nuclear abundance of NRF2. However, overexpressing TNF-*α* reverses the inhibition of PDTC on the NRF2 expression and its nuclear abundance, and knockdown of proinflammatory cytokines (TNF-*α*, IL-1*β*, IL-6, or IL-8) significantly decreases the LPS-induced NRF2 expression and the nuclear abundance of NRF2. Thus, the results from this study suggest that the interaction between NRF2 and NF-*κ*B is not a simple upstream and downstream relationship, and the activation of NRF2 is dependent on proinflammatory cytokines in LPS-induced gEECs inflammation. This data conflicts with data described in previously studied inflammation models [25, 26].

Overall, our results suggest that proinflammatory cytokines induced by LPS through the TLR4/NF-*κ*B pathway promote the NRF2 expression and its translocation into the nucleus. In the nucleus, NRF2 inhibited the expression of proinflammatory cytokines by directly binding to p65. At the same time, it must be emphasized that the NF-*κ*B inhibitor reduces LPS-induced NRF2 expression and nuclear abundance of NRF2, which differs from a previously studied inflammation model. However, it should be noted that our research still has many shortcomings, such as the molecular mechanisms of how LPS stimulates the activation of NRF2 (for example via promoting the degradation of Keap1, by protecting Nrf2 stability against ubiquitination and/or proteasomal degradation) remain unclear and need to be addressed. Therefore, more data are needed to address these questions in future studies.

## Figures and Tables

**Table 1:** Primers used in RT-qPCR, ChiP analysis, and si-RNA sequence.

Primers used in RT-qPCR analysis	
TNF-*α*	Forward: 5′-ATAACAAGCCGGTAGCCCAC-3′
	Reverse: 5′-CTCAGTCTGGATTCAGCCCC-3′
IL-1*β*	Forward: 5′-CGCATGTTCCTGGGGAGATT-3′
	Reverse: 5′-TGGGATGCAACATGGCTCTT-3′
IL-6	Forward: 5′-GCTTCTGCATTGGGAGGTCT-3′
	Reverse: 5′-ACAGGACATAGTCTGCCCCT-3′
IL-8	Forward: 5′-CATGCCTGGATAGCAAGCCT-3′
	Reverse: 5′-TGATAGCTGCCTGAAGCTCG-3′
*β*-Actin	Forward: 5′-GGCTGTATTCCCCTCCATCG-3′
	Reverse: 5′-CCAGTTGGTAACAATGCCATGT-3′

Primers used in ChiP analysis	
TNF-*α*	Forward: 5′-GAGGCAATAGGTTTTGAGG-3′
	Reverse: 5′-AAGCATCAAGGATACCCTC-3′
IL-1*β*	Forward: 5′-ACCCTCACCCTCCAACAAAG-3′
	Reverse: 5′-TGGAAGGGCAAGGAGTAGCA-3′
IL-6	Forward: 5′-GTGTCTTCCACTTTGTCCCACA-3′
	Reverse: 5′-TGGAAGGGCAAGGAGTAGCA-3′
IL-8	Forward: 5′-GGCTTCCCTGATAGCTCAGTT-3′
	Reverse: 5′-TGAACCCAGGTCTACCCACAT-3′

si-RNA sequence	
si-NC	Forward: 5′-AAGUUCAGGACUAAGUCAGGC-3′
	Reverse: 5′-GAGACUAUGACUCGUAAUUAC-3′
si-TNF-*α*	Forward: 5′-UCUUUCUCUCUCAUUUCUCUC-3′
	Reverse: 5′-GAGAAAUGAGAGAGAAAGAGG-3′
si-IL-1*β*	Forward: 5′-ACUGUAAUGAAAACAGAUGUG-3′
	Reverse: 5′-CAUCUGUUUUCAUUACAGUGA-3′
si-IL-6	Forward: 5′-AAGGAUUUCCUUCACUUACUU-3′
	Reverse: 5′-GUAAGUGAAGGAAAUCCUUAG-3′
si-IL-8	Forward: 5′-AUUAGAAGAAUAUGCUUACCU-3′
	Reverse: 5′-GUAAGCAUAUUCUUCUAAUCU-3′

## Data Availability

The datasets used and/or analyzed during the current study are available from the corresponding author on reasonable request.

## Supplementary Materials

Supplementary Materials Figure S1: depleting NRF2 increased LPS-induced proinflammatory or anti-inflammatory-related gene expression. Control and NRF2 knockout (KO) gEECs were stimulated with or without 8 *μ*g/mL LPS for 12 hours and then RT-qPCR analysis of the indicated iNOS, COX-2, HO-1, and NQO1 mRNA expression. Data shown are mean ± SD; ns was *P* > 0.05, and ### was *P* < 0.001 vs. control group; ^∗^ was *P* < 0.05, ^∗∗^ was *P* < 0.001, and ^∗∗∗^ was *P* < 0.001 vs. WT group. *P* value was calculated by the one-way ANOVA with the Tukey's test as a posthoc test.
