# Supplementary material for: NRF2 Exerts Anti-Inflammatory Effects in LPS-Induced gEECs by Inhibiting the Activation of the NF-κB
Source: Mediators Inflamm. 2021 Nov 2;2021:9960721. doi: 10.1155/2021/9960721 (PMC8577927; doi:10.1155/2021/9960721)

**Figure S1** Depleting NRF2 increased LPS-induced pro-inflammatory or anti-inflammatory-related gene expression. Control and NRF2 knockout (KO) gEECs were stimulated with or without 8 μg/ml LPS for 12 hours, and then RT-qPCR analysis of the indicated iNOS, COX-2, HO-1 and NQO1 mRNA expression. Data shown are mean ± SD; ns was P>0.05 and ### was P<0.001 vs control group, * was P<0.05, ** was P<0.001 and *** was P<0.001 vs WT group. P value was calculated by the one-way ANOVA with the Tukey’s test as a post hoc test.


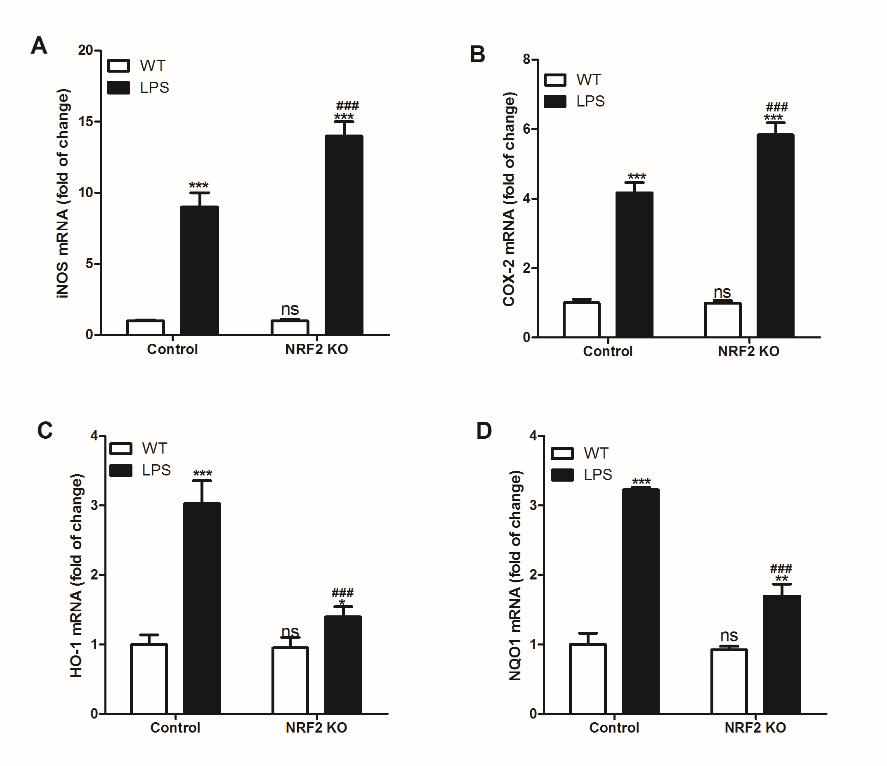

Supplement: Supplementary Materials — Figure S1: depleting NRF2 increased LPS-induced proinflammatory or anti-inflammatory-related gene expression. Control and NRF2 knockout (KO) gEECs were stimulated with or without 8 μg/mL LPS for 12 hours and then RT-qPCR analysis of the indicated iNOS, COX-2, HO-1, and NQO1 mRNA expression. Data shown are mean ± SD; ns was P > 0.05, and ### was P < 0.001 vs. control group; ∗ was P < 0.05, ∗∗ was P < 0.001, and ∗∗∗ was P < 0.001 vs. WT group. P value was calculated by the one-way ANOVA with the Tukey's test as a posthoc test. [file 9960721.f1.docx]
